# The Immunohistochemical Expression of MCM-3, -5, and -7 Proteins in the Uterine Fibroids

**DOI:** 10.3390/cimb43020058

**Published:** 2021-07-25

**Authors:** Piotr Rubisz, Lidia Hirnle, Christopher Kobierzycki

**Affiliations:** 1 Gynecological Surgery, Endometriosis Treatment Center, Medicus Hospital, 50-224 Wroclaw, Poland; piotr.rubisz@gmail.com; 2 First Department of Gynecology and Obstetrics, Wroclaw Medical University, 50-368 Wroclaw, Poland; lidia.hirnle@umed.wroc.pl; 3 Division of Histology and Embryology, Department of Histology and Human Morphology, Wroclaw Medical University, 50-368 Wroclaw, Poland

**Keywords:** minichromosome maintenance protein, MCM, uterine fibroids, myoma, leiomyoma, immunohistochemistry

## Abstract

Uterine fibroids are the most common mesenchymal uterine neoplasms; their prevalence is estimated in 40%–60% of women under 35 and in 70%–80% of women over 50 years of age. The current research aims to focus on the etiopathogenesis of uterine fibroids, the factors that affect their growth, and markers with diagnostic and prognostic properties. The MCM (minichromosome maintenance) protein family consists of peptides whose primary function is participation in the molecular mechanism of creating replication forks while regulating DNA synthesis. The aim of this work was to determine the proliferative potential of uterine fibroid cells based on the expression of the Ki-67 antigen and the MCMs—i.e., MCM-3, MCM-5, and MCM-7. In addition, the expression of estrogen (ER) and progesterone (PgR) receptors was evaluated and correlated with the expression of the abovementioned observations. Ultimately, received results were analyzed in terms of clinical and pathological data. Materials and methods: In forty-four cases of uterine fibroids, immunohistochemical reactions were performed. A tissue microarray (TMA) technique was utilized and analyzed cases were assessed in triplicate. Immunohistochemistry was performed using antibodies against Ki-67 antigen, ER, PgR, MCM-3, MCM-5, and MCM-8 on an automated staining platform. Reactions were digitalized by a histologic scanner and quantified utilizing dedicated software for nuclear analysis. Assessment was based on quantification expression of the three histiospots, each representing one case in TMA. Results: In the study group (uterine fibroids), statistically significant stronger expression of all the investigated MCMs was observed, as compared to the control group. In addition, moderate and strong positive correlations were found between all tested proliferative markers. The expression of the MCM-7 protein also correlated positively with ER and PgR. With regard to clinical and pathological data, there was a negative correlation between the expression of MCMs and the number of both pregnancies and births. Significant reductions in MCM-5 and MCM-7 expression were observed in the group of women receiving oral hormonal contraceptives, while smoking women showed an increase in MCM-7, ER, and PgR. Conclusions: Uterine fibroid cells have greater proliferative potential, as evaluated by expression of the Ki-67 antigen and MCMs, than unaltered myometrial cells of the uterine corpus. The expression of MCM-7 was found to have strong or moderate correlations in all assessed relations. In the context of the clinical data, as well evident proliferative potential of MCMs, further studies are strongly recommended.

## 1. Introduction

### 1.1. Epidemiology

Benign tumors originating in the muscle layer of the uterine wall (uterine fibroids) are the most common neoplasms in the female population [1]. They are found clinically in about 25% of all examined patients [2]. In women under 35, incidence is estimated between 40–60%, and it increases with age, even reaching 70–80% in women over 50 [3]. They are found in as many as 77% of resected uteri [4]. A significant proportion of uterine fibroids remains asymptomatic, which additionally makes it impossible to accurately assess their frequency. However, their presence was not found before pubarche, and their incidence increases with the patient’s age during the reproductive period [3,5]. They do not grow in the postmenopausal period, with observed regress over time in some cases. Despite the common occurrence of the described neoplasms, their etiopathogenesis is still unclear [6].

### 1.2. Risk Factors

The occurrence of uterine fibroids is 2–3 times more frequent in females of the black race compared to the white race [3,5]. Moreover, in females of the black race, they occur at a younger age and have a more severe clinical course [7]. In them, the symptoms of uterine fibroids appear on average 6 years earlier, even at around 20 years of age. In the white race, symptoms most often appear in the age range of 30-40 years [8]. In the yellow race, the incidence of these tumors is similar to that in the white race [5]. Early onset of menstruation is associated with a higher risk of uterine fibroids. This may be due to a greater exposure to the number of ovulatory cycles and therefore a longer exposure to ovarian hormones [8,9,10,11]. What also interferes with this observation is the fact that uterine fibroids are less common in women who have given birth [10,11,12,13]. The more frequent occurrence of uterine fibroids in nulliparous women is most likely related to the constant estrogen exposure, uninterrupted by lactation, and the accompanying greater number of menstrual cycles [14]. Many studies have highlighted the fact that a later age at the time of the first pregnancy was associated with a lower risk of uterine fibroids [15,16]. The available literature has consistently shown that the later the age from the last pregnancy, the greater the risk of uterine fibroids [13,16,17]. Interestingly, there are divergent opinions regarding the influence of exogenous hormones on the formation and growth of uterine fibroids. Wise et al. did not show the effect of the hormonal preparations used on the incidence of uterine fibroids throughout life. It has only been found that early initiation of hormonal contraception (HC) is associated with a higher risk of uterine fibroids [17]. Similarly, Terry et al. and Samadi et al. found no effect of the use of oral contraceptive pills (OC) on the occurrence of uterine fibroids [12,18]. In the available literature, we also find reports on a reduced risk of uterine fibroid development with the use of HC. Ross et al. found that taking contraceptive preparations reduces the risk of uterine fibroids, which also decreases with the duration of HC intake [16]. The protective effect of AH was also confirmed by Lumbinganon et al. In an analysis of 910 cases of operated uterine fibroids, the protective effect of HC and DMPA (a form of medroxyprogesterone acetate depot) was demonstrated. Like Ross et al., they confirmed the relationship between the duration of hormone therapy use and a reduced risk of uterine fibroids (even 90% with >5 years of DMPA use) [10]. On the other hand, Marshall et al. found a higher risk of uterine fibroids if HC was started at a young age (13–16 years)—relative risk (RR) 2.29 (1.56–3.36) [11]. The increased incidence of uterine fibroids in relation to body weight has not been thoroughly documented. According to some researchers, there is a positive correlation with the occurrence of uterine fibroids with the increase in body mass expressed by the body mass index (BMI) [10,15,19]. According to Wise et al., in patients with a BMI above 28, an increase in the risk of uterine fibroids was observed at the level of over 50%, while the average population risk in the study group was 7.2% [17]. Additionally, Wise et al. demonstrated a threefold increase in the risk of uterine fibroids in women with a body weight over 70 kg compared to women with a body weight of 50–60 kg [17]. However, the available literature also includes studies contradicting the influence of body weight on the incidence of uterine fibroids [13]. Alcohol consumption increased the overall risk of uterine fibroids in the studied women [20,21]. Alcohol increases the level of estrogen in the blood regardless of the phase of the menstrual cycle, which may explain its positive effect on the growth of uterine fibroids [22]. Many studies have found a reduced risk of uterine fibroids in smokers [23,24]. According to some researchers, the risk of uterine fibroids in this group is even 20-50% lower than in non-smokers [23]. Lower levels of estrogens were found in smoking women, which is most likely due to aromatase inhibition by components of tobacco smoke [25]. Perhaps such less active forms of estrogen may reduce the incidence of uterine fibroids in group of smoking respondents [26]. In the available literature, there were also works that denied the relationship between cigarette smoking and uterine fibroids [15,19,21].

### 1.3. MCM Proteins

The family of minichromosome maintenance proteins (MCM) was first identified in Saccharomyces cerevisiae cells. The primary function of MCM proteins is to control cell division through replication licensing. At present, we know ten MCM proteins marked with numbers from 1 to 10; however, the MCM protein family includes only eight of them (MCM 2–9). The MCM-1 and MCM-10 proteins do not have a specific MCM domain and therefore do not belong to the family [27]. The MCM-3 protein acts as an inhibitor of cell division, inhibiting the transition of cells to the S phase. The inhibitory capacity is most likely due to post-translational modifications [28]. According to Lee et al., the MCM-3 protein is more useful in determining the proliferation potential of cells than the well-known and recognized marker of cellular proliferation Ki-67 in thyroid cancer [29]. A correlation was also found between increased expression of the MCM-3 protein and a worse prognosis in patients with gliomas [30], tumors of the salivary glands [31], thyroid gland [29], and melanomas [32]. Kobierzycki et al. found a strong positive correlation between the expression of the MCM-3 protein and the Ki-67 antigen, with the increase in the degree of malignancy in ovarian cancer [33]. The MCM-5 protein plays a known role in the transition of a cell from the G1 to the S phase. Additionally, being a component of the MCM 2-7 complex, it coordinates the process of replication activation and participates in the formation of replication forks [34]. Many studies confirm that MCM-5 protein expression is a good marker of cell proliferation in malignant lesions such as bladder cancer and gastrointestinal neoplasms, including esophageal cancer [35]. Recent studies have shown that the level of MCM-5 and CDC6 expression positively correlates with the severity of cervical dysplasia [36]. According to Shang-Yang Yu et al., the increased level of MCM-5 protein expression strongly positively correlates with the aggressive course and poor prognosis in squamous cell carcinoma of the oral mucosa [37]. The MCM-7 protein, due to its slightly different structure, has the ability to combine with proteins regulating the cell cycle such as: pRb [38], Mat-1 [39], FHL2 [40], and the viral HPV-E6 oncoprotein [41]. The functions of the MCM-7 protein in regulating the cell cycle and DNA proliferation can be modulated by oncogenes such as Ras and MYCN (and C-MYC) [27]. Many studies have found an increased expression of MCM-7 protein in malignant neoplasms such as prostate cancer [42], head and neck cancer [43], cervix [44], and pancreatic cancer [45]. MCM-7 protein overexpression is observed during HPV infection. It can be both a good indicator of E7 oncoprotein activity and a marker of its induced DNA synthesis in the HPV life cycle [41,44]. Due to the fact that expression of the MCM-7 protein may be increased independently of other MCM proteins in tumor cells, further detailed analysis of the function of this protein in the regulation of the cell cycle is necessary.

### 1.4. Aim of the Study

Despite many known and studied markers of cellular proliferation, it is necessary to constantly search for more sensitive and specific markers that would enable not only an accurate differential diagnosis of the studied changes, but also an understanding of the mechanisms controlling the cell cycle, as well as their effective treatment and prevention. Scientific research confirms the greater specificity of MCM proteins as markers of cell proliferation compared to the Ki-67 antigen, which is the most studied marker [42,44]. The main advantage of MCM proteins, unlike Ki-67, is the exact knowledge of the functions performed by these proteins in the cell, i.e., primary involvement in DNA replication. Currently, in one of the largest medical databases containing a list of works in the fields of medicine, biology, and related sciences, PubMed, there is only one two paper referring to the topic of MCM expression in leiomyomas [46,47]. The aim of the study was to determine the proliferation potential of uterine fibroids based on the expression of the commonly determined Ki-67 proliferative antigen and the tested MCM proteins, i.e., MCM-3, MCM-5 and MCM-7. Moreover, the expression of estrogen and progesterone receptors was assessed and the obtained results were correlated with the expression of the above-mentioned markers. Finally, the expression of all tested markers was analyzed in terms of the clinical and pathological data.

## 2. Materials and Methods

### 2.1. Patient Characteristics

All operated patients were Caucasians. The mean age of the operated patients was 46.0 (±9.2) years. Body mass index (BMI) ranged from 18.4 (underweight) to 35.6 (obesity stage II–clinical obesity), with a median of 25.0 (±3.7) kg. The basic clinical features of the studied patients are presented in Table 1. Both nulliparous and multiparous women were included in the study. The survey included the most common complaints and symptoms related to the occurrence of uterine fibroids (pressure on the bladder, pollakiuria, anemia, infertility lasting over 12 months). Additionally, the presence of the most common known factors that may correlate with the presence and influence the etiology of uterine fibroids, such as nicotinism, recurrent infections of the reproductive organ, and combined hormonal contraception, was verified. Table 2 presents the percentage characteristics of the qualitative traits in the studied group of patients with uterine fibroids.

In addition to the pain related to the presence of uterine fibroids reported by patients, 43% experienced dysuria. Over 40% of the patients reported pressure on the urinary bladder and pollakiuria, which may be directly related to the increase in uterine dimensions in the course of the occurrence of fibroids in patients. Due to increased uterine bleeding, 31.8% of the subjects had a history of anemia, which required pharmacological treatment in each of the presented cases. In 9.1%, it was necessary to administer blood products to normalize the parameters of the red blood cell system. Among the known risk factors for uterine sarcomas, the operated patients reported nicotinism (18.2%) and recurrent pelvic infections (9.1%). Based on a detailed interview, as many as 36.4% of the surveyed women had a family history of uterine fibroids. Most often, in as many as 70.5% of cases, uterine fibroids occurred in the mothers of the patients, but less often in the sisters (9.15%) and grandmothers (6.8%). The number and size of uterine fibroids were assessed macroscopically postoperatively and the results were compared with the histopathological examination. Similar values were obtained. Moreover, Table 1 shows the size and number of uterine fibroids. The mean number of uterine fibroids in the operated women was 2.5, while the mean size of the operated lesions was about 5.3 cm. The study group of women underwent a questionnaire study to assess uterine bleeding. The severity of menstrual pain was measured using a numeric rating scale (NRS). The NRS scale includes 11 pain levels–from 0 to 10, where 0 is no pain at all and 10 is the worst imaginable. Similarly, the above scale was used to assess the degree of pressure on the bladder by the myomatoid uterus. In the studied group of patients, regular menstrual cycles occurred in 81.8% of women. In 13 out of 44 examined patients, the hemoglobin level before surgery was below 12 g/dl, which may be related to the profuse menstrual bleeding and the presence of blood clots, which was reported in 84.1% of cases. Due to the pain complaints occurring in patients both during and outside menstruation (70.5% vs. 31.8% of patients), as many as 47.7% of women regularly used painkillers. Of these, the most commonly used was Ibuprofen. Due to the presence of uterine fibroids, more than a quarter of the surveyed patients experienced permanent pain in the lower abdomen that was not related to menstruation. 

### 2.2. Tissue Obtaining

The study included 50 patients undergoing total or supracervical amputation of the uterus, as well as patients qualified for the procedure of enucleation of uterine fibroids. Of these, 44 permanently menstruating patients were included in the study. The material collected intraoperatively was placed in 4% buffered formalin and immediately forwarded for routine histopathological evaluation. In each of the operated patients, a fragment of uterine fibroid tissue was collected from the material obtained during the procedure—the study group; and a fragment of tissue from the macroscopically unchanged uterine muscle—the control group (subsequently microscopically verified).

### 2.3. Tissue Microarray Preparation (TMA)

All available tissue material underwent routine FFPE procedure. was secured in the form of paraffin blocks. On 6 µm paraffin sections, a topographic hematoxylin and eosin (HE) staining was performed as usual. In the case of 44 specimens suspected of “uterine fibroids”, the initial diagnosis was verified, while 44 cases of tissue surrounding the tumor were analyzed to exclude the presence of abnormal microarchitecture of the uterine wall muscle tissue. Assessment was performed using a BX41 light microscope (Olympus, Japan). The experiment included all 44 cases of uterine fibroids and 5 cases of normal, representative muscle tissue around the tumor. HE stained sections were digitized using a Pannoramic MIDI II histology scanner (3DHistech, Hungary). Then, for each case, 3 locations were selected on a section with a representative tissue structure. Tissue microarrays were prepared with the presence of 147 histiospots (132 = 44 × 3 for the test group and 15 = 5 × 3 for the control group) using the TMA Grand Master automatic tissue microarray preparation equipment (3DHistech) with a 2 mm collection needle. The tissue microarray blocks prepared in this way were used to prepare typical paraffin sections for further stages of the study.

### 2.4. Immunohistochemistry (IHC)

All reactions were performed on 4 μm-thick paraffin sections. For deparaffinization, hydration, and exposure of antigenic determinants, the sections were boiled in Target Retrieval Solution High pH buffer (for antibodies against ERα, PgR, MCM-3, MCM-5, MCM-7) and Target Retrieval Solution Low pH (for Ki-67) in a PTLink apparatus (20 min. 97 °C), then cooled in TBS washing buffer with 0.1% Tween-20. The IHC reaction was performed using the EnVision FLEX visualization system and the AutostainerLink 48 automated platform (Dako, Glostrup, Denmark). The first step was to block endogenous peroxidase by incubation for 5 min. at EnVision FLEX Peroxidase Blocking Reagent (Dako). The sections were then washed twice with TBS with 0.1% Tween-20 and incubated for 20 min. with primary monoclonal antibodies: ER (RTU—ready-to-use, IR084, Dako), PgR (RTU, IR068, Dako), MCM-3 (1:100, M7263, Dako), MCM-5 (1:100, sc-165994, Santa Cruz Biotechnology,), MCM-7 (1:50, sc-9966, Santa Cruz Biotechnology), and Ki-67 (RTU, IR626, Dako). Excess antibodies were removed by washing twice with TBS with 0.1% Tween-20 and secondary antibodies labeled with horseradish peroxidase (EnVision FLEX HRP) were applied and incubated for 20 min. Sections were washed twice with TBS with 0.1% Tween-20. In the next step, the substrate for horseradish peroxidase was added—diaminobenzidine (EnVision FLEX)— and incubated for 10 min. Reagent residues were removed by washing twice with TBS with 0.1% Tween-20. Subsequently, counterstaining with hematoxylin (EnVision FLEX Hematoxylin) was performed and incubated for 5 min. After rinsing in distilled water, the sections were dehydrated by carrying out a series of dilutions with ethyl alcohol (70%, 96%, 99.8%) for 5 min at each concentration, followed by xylene (5 min). Slides were closed with mounting medium synthetic resin in a Coverslipper (Dako).

### 2.5. Evaluation of Reactions

Immunohistochemical reactions were assessed using Quant Center software with a Nuclear Quant module (3DHistech) for the analysis of nuclear reactions. For each of the 3 histiospots representing one TMA case, the percentage of cells expressing the marker under investigation was determined in relation to all altered cells. The final score for each case was the mean value of the 3 histiospots.

### 2.6. Statistical Analysis

STATISTICA v.12 (StatSoft, Inc., Tulsa, OK, USA) and MS Excel spreadsheet (Microsoft Windows, Albuquerque, NM, USA) were used in the statistical analysis. For all quantitative parameters (age, body weight, BMI, etc.), their distribution was checked for compliance with the normal distribution. Conformity was assessed with the Shapiro–Wilk test. *p* < 0.05 was adopted as the critical level of significance. The significance of differences in mean values in two independent groups (place of taking the slice) for parameters with a distribution significantly deviating from the normal or with heterogeneous variances was checked using the Mann–Whitney U test. Spearman’s rank correlation coefficient (r) was calculated to analyze the relationships between the variables measured on an ordinal scale (e.g., pain intensity). Correlation analysis was used to establish and evaluate the relationship between the expression of the studied markers and the somatic and clinical features of the studied patients. The test results were considered statistically significant for *p* < 0.05.

## 3. Results

**Figure 1:**
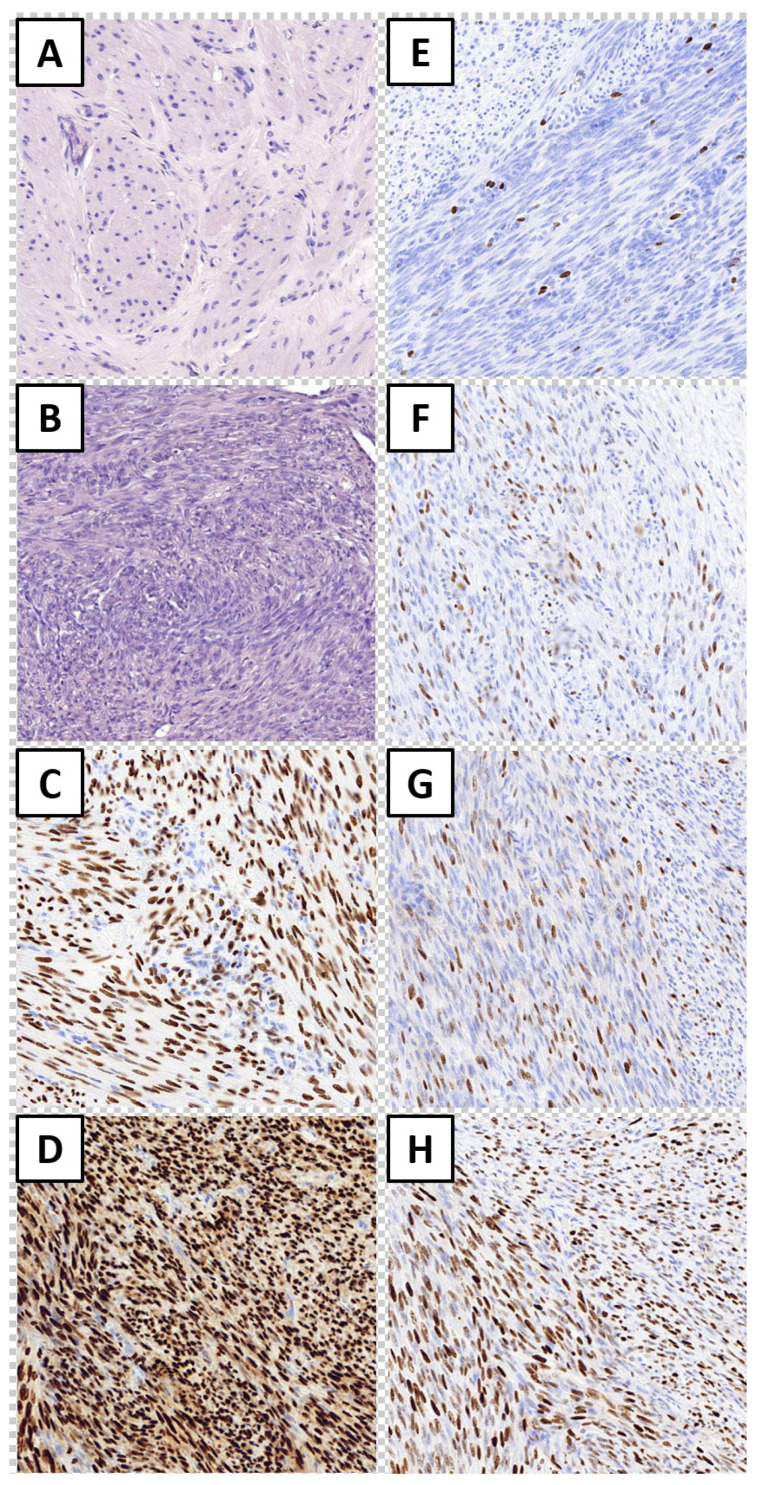
Hematoxylin and eosin staining of healthy myometrium (**A**) and leiomyoma (**B**). Immunohistochemical expression of estrogen receptor (**C**), progesterone receptor (**D**), Ki-67 antigen (**E**), MCM-3 (**F**), MCM-5 (**G**), and MCM-7 (**H**). Magnification ×200.A weaker expression of MCM-5 was observed in the cases of women using DTA (*p* = 0.034); however, in those patients who underwent conservative treatment of uterine fibroids (ulipristal acetate), MCM-5 expression was stronger (*p* = 0.04).

On all available cases, HE staining was initially performed to reevaluate material into healthy myometrium—control group (Figure 1A) and uterine fibroid—study group (Figure 1B). In conducted immunohistochemical reactions, nuclear expression of all tested markers was observed (Figure 1C, H). Significantly stronger expression of tested markers in the study group was disclosed for PgR (89.3% vs. 70.8%; *p* = 0.009,) MCM-3 (1.6% vs. 0.2%; *p* = 0.033). MCM-5 (4.2% vs. 0.8%; *p* = 0.021) and MCM-7 (32.8% vs. 8.4%; *p* = 0.014; Mann–Whitney for all). In regard to ER and Ki-67 expression, differences between study and control groups were insignificant (*p* = 0.129 and *p* = 0.399, respectively). 

The expression of MCM-7 correlated with all tested markers, whereas expression of Ki-67 correlated with all tested MCM proteins (Spearman correlation test). All disclosed associations are presented in the table. In bold as the relationships that reached a level of statistical significance. The expression of studied markers was assessed in regard to quantitative and qualitative variables from medical records of study group patients. All associations tested by Spearman correlation assay are presented in Table 3. Statistically significant associations are given in bold and, for qualitative characteristics, subsequent Mann–Whitney tests were performed. The expression of ER was stronger in smokers (87.3% vs. 57.3%; *p* < 0.001), and increased intake of cigarettes per day affected the increase in expression of ER (r = +0.588; *p* < 0.001). Moreover, it was disclosed that maternal age of first pregnancy negatively correlated with ER expression, i.e., older primiparas presented decreased expression of ER (r = −0.355; *p* < 0.05). Similarly, the expression of PgR was stronger in smokers (96.9% vs. 87.4%; *p* = 0.004), and increased intake of cigarettes per day affected the increase in expression of PgR (r = +0.453; *p* < 0.01). Additionally, statistically more frequent PgR expression was found in cases of familial uterine fibroids (*p* = 0.008). PgR expression was stronger in women with a history of uterine fibroids, especially in relation to sisters (*p* = 0.015). Ki-67 expression was stronger in women with a history of uterine fibroids, especially in relation to grandmothers (*p* = 0.032).

A weaker expression of MCM-3 was observed with an increase in the number of pregnancies (r = −0.309; *p* < 0.05) and the number of deliveries (r = −0.308; *p* < 0.05) in the patients. Similarly, a weaker expression of MCM-5 was observed with an increase in the number of pregnancies (r = −0.459; *p* < 0.01) and the number of deliveries (r = −0.434; *p* < 0.01) in the patients.

The expression of MCM-7 was stronger in smokers (49.3% vs. 28.9%; *p* = 0.01), and increased intake of cigarettes per day affected the increase in expression of MCM-7 (r = +0.402; *p* < 0.01). A weaker expression of MCM-7 protein was observed with an increase in the number of pregnancies (r = −0.310; *p* < 0.05) and the number of deliveries (r = −0.320; *p* < 0.05) in the patients. Moreover, a weaker expression of MCM-7 was observed in the cases of women using DTA (*p* = 0.004).

## 4. Discussion

Since the 1990s, the immunohistochemical technique has been used for the differential diagnosis of neoplastic changes in uterine smooth muscles to assess the expression of proliferative markers, e.g., Ki-67 antigen, p53 protein, PCNA, as well as expression of steroid hormone receptors, i.e., ER and PgR. Pathologists agree that classical uterine fibroids are characterized by weak Ki-67 and p53 protein expression, as well as strong ER and PgR expression. As the expression of markers of cellular proliferation increases, the risk of neoplastic transformation of a lesion increases [48,49]. Additionally, decreased expression of ER and PgR is specific for aggressive lesions. Despite the small number of studies, it is estimated that expression >10% for ER and PgR and weak Ki-67 expression is a favorable prognostic factor in patients with LMS [50]. In our study, the expression level of ER and PgR, as well as the Ki-67 proliferative antigen in uterine fibroids and in the normal muscle layer of the uterus, was determined. The study group showed a stronger expression of ER compared to the control group (60.4% vs. 47.2%; *p* = 0.129); however, this difference did not reach statistical significance. For PgR expression, there was a significant statistical difference between the study group and the control group (89.2% vs. 70.8%; *p* = 0.009). The obtained results are similar to the results of numerous studies confirming the high expression of ER and PgR in uterine myoma cells [50,51,52]. The conducted analyses confirmed the fact of low expression of the Ki-67 marker in uterine myomas, as known from other studies. The difference in expression between uterine myoma cells and cells of the normal muscle layer was found to be statistically insignificant (0.5% vs. 0.2%; *p* = 0.399). Similar results can be found in the studies of Amada et al., where a weak expression of the Ki-67 protein was described (only in 1/24 of cases did this expression exceed 3.6% of uterine fibroids) [48]. On the other hand, according to Mittal et al. only 1 out of 15 examined leiomyomas showed the expression of Ki-67 at the level of 5–10% [51]. At present, despite the use of well-known markers in the differential diagnosis, including Ki-67 and steroid receptors ER and PgR, it is justified to search for new, more sensitive and specific markers of benign lesions originating from the muscle layer of the uterine wall. It is extremely important to find markers specific for a given type of lesions and those that can be used to estimate the possible progression and determine the advancement of the disease. High hopes are currently placed in the proteins of the MCM family. They are factors that initiate and limit DNA replication [53]. MCM proteins are extremely stable and simultaneously present during all phases of G1, S, G2, and M. As the cell goes into a resting phase, differentiation or aging is not observed. The Ki-67 antigen used in diagnostics until how is detected not only in the phases of cell division, but also in other phases of the cell cycle [27]. MCM proteins are not detected in cells a few days after passing the resting or differentiated phase [27]. This property appears to be diagnostically useful [54]. Considering the above, it was found that cells of normal tissues that have already undergone the process of differentiation, e.g., keratinocytes of the skin surface layer, show poor expression of MCM proliferative proteins. On the other hand, the basal and basal layer cells, due to the possibility of further divisions, express MCM proteins higher than keratinocytes [55]. Studies by Freeman et al. showed that the expression of MCM proteins in normal squamous epithelium remains at a similarly low level. For MCM-5 protein, it was approximately 15% for cervical, pharyngeal, skin, and esophagus epithelium. Differential expression of MCM-2 and MCM-5 proteins was demonstrated for glandular epithelial cells. It was the highest for the glandular epithelium of the large intestine, approximately 50%. The lowest expression of MCM-2 and MCM-5 proteins was found in normal kidney parenchyma (about 2%) and bladder (17–20%) cells [56]. Our own study similarly confirmed the poor expression of MCM proteins in the examined tissues of the control group—the normal muscle layer. It was 0.2% for MCM-3 protein, 0.8% for MCM-5, and 8.4% for MCM-7 protein. The obtained results prove the low proliferation potential of the normal tissue of the uterine muscle. In the available literature, only one study analyzed the expression of MCM proteins in cells of the muscle layer and uterine fibroids. Examining the MCM-7 protein, Chuang et al. showed low expression in normal uterine muscle cells compared to higher expression in leiomyoma cells (*p* < 0.01) [46]. Many researchers have confirmed the increased expression of MCM proteins in pre-neoplastic lesions and benign neoplasms. It was observed that the expression of MCM proteins does not depend on the presence of inflammation. Freeman et al. did not show the expression of MCM proteins in cases of inflammation of kidney tissues and a comparable expression of MCM in cases of normal and inflamed tissues taken from the bladder. On the other hand, in cervical epithelial cells, a positive correlation was found between the expression of MCM-5 and MCM-2 proteins and the severity of dysplasia [56]. Similarly, Mukherjee et al. indicated an increase in MCM expression in cervical dysplastic lesions, which, combined with Papanicolaou (Pap) cytological evaluation of cell smears, enabled more sensitive and specific results in the diagnosis of cervical precancerous lesions [57]. Taking into account the above application of the evaluation of MCM protein expression, it may turn out to be extremely useful in differentiating dysplastic changes from reactive changes in tissues, which often creates a diagnostic problem using only the morphological criteria of the examined tissues. To date, there are no studies comparing the expression of MCM proliferative proteins in uterine fibroids in the available literature. In our own research, the expression level of MCM-3, MCM-5 and, MCM-7 proteins in neoplastic tissues was determined. Significantly higher expression (expressed as a percentage of stained cells) was observed in the group of tissues collected with leiomyoma than in the group of tissues taken from the muscle layer for each of the examined MCM proteins (*p* < 0.05). The highest percentage of stained cells (32.8%) in uterine fibroids was found for MCM-7 protein expression. Correspondingly, the value for MCM3 was 1.6% and was 4.2% for MCM-5. These studies confirm the fact of higher expression of MCM proteins in benign and dysplastic lesions, known from the literature, in relation to the examined normal tissues [55,56,57]. Moreover, it is known that the appearance of positive MCM cells can be correlated with an increase in the number of proliferating cells, as well as with a decrease in the histological degree of differentiation of a neoplastic lesion [44]. Therefore, in the case of malignant lesions, e.g., originating from the epithelium of the cervix [36], oral cavity [37], larynx [58], or urinary tract [59], higher expression of MCM proteins was shown compared to normal tissues. Freeman et al. additionally showed that the expression of MCM proteins increased with a decrease in the degree of differentiation of lesions in malignant tumors of the skin, bladder, and colon. Moreover, they found higher expression expressed as the percentage of positively stained cells in the above tumors for the MCM-5 protein compared to PCNA and Ki-67 [57]. Although PCNA and Ki-67 are recognized markers of cell proliferation, they do not fully define the stages of the cell cycle, as PCNA expression is also increased in cells during DNA repair, and Ki-67 is not the major determinant of cell proliferation [60]. Many authors find a positive correlation between the expression of Ki-67 and MCM in neoplastic lesions. Nowińska et al., found a statistically significant positive correlation between the expression of each of the examined proteins MCM-2, MCM-3, and MCM-7 with Ki-67 in squamous cell carcinomas of the larynx. Moreover, this study showed positive correlations between the expression of each of the tested MCM proteins [58]. In our research we obtained similar results. The strongest positive correlation was observed for the MCM-3 and MCM-5 proteins (r = 0.727). Similarly, a positive correlation occurred for the expression of MCM-3 and MCM-7 proteins (r = 0.674) and for the expression of MCM-5 and MCM-7 proteins (r = 0.687). This fact may be related to the partially common mechanism of action of MCM proteins in the form of a complex of MCM-2-7 proteins. It is only through the joint action of the MCM protein hexamer and many other regulatory proteins that the DNA replication process necessary for tumorigenesis is possible. It is also worth noting that the expression of each of the examined MCM proteins positively correlated with the expression of the Ki-67 marker, despite the fact that the latter shows a weak expression in uterine fibroids, which is also confirmed by the previously quoted data from the literature. The weakest correlation was demonstrated for the MCM-7 protein (r = 0.313) and the strongest for the MCM5 protein (r = 0.579). In the presented cases, the expression of the studied markers was analyzed depending on the factors that might affect the etiology of uterine fibroids. Taking into account the known and documented risk factors for the development of uterine fibroids, the relationship between the number of pregnancies and deliveries and the expression of MCM proteins in uterine fibroids deserves special attention. As discussed earlier, according to many authors, the incidence of uterine fibroids decreases with the number of pregnancies and deliveries [10,11,17]. Wise et al. and Chen et al. found that the relatively constant concentration of estrogens during the cycle and the associated higher estrogen exposure in women, without interruptions in exposure during pregnancy and childbirth, are the causes of higher uterine fibroids in nulliparous women [14,17]. Perhaps it is related to the results obtained in our material because the expression of each of the examined MCM proteins is negatively affected not only by the number of pregnancies but also by the number of deliveries (*p* < 0.05). It is possible that reduced expression of MCM proteins involved in DNA replication leads to inhibition of tumorigenesis of uterine fibroids and inhibition of transformation of normal myometrium into leiomyoma cells. However, the presented study does not confirm the reduced expression of MCM proteins depending on age at the time of the first pregnancy and the number of years since the last birth, which, according to many authors, affects the occurrence of uterine fibroids [13,16,17]. Neiger et al. demonstrated a reduced expression of estrogen receptors and a reduced sensitivity of uterine fibroids to estrogens in pregnancy [61]. In 2006, Pan et al. described a number of mechanisms in which progesterone and estrogen can modify the regulation of DNA replication by affecting the MCM protein complex [62]. In this study, there was no effect of progesterone on the expression of MCM-7 protein only, which may be related to the results obtained in the study. Additionally, in our own study it was found that only in the case of the MCM-7 protein is there a statistically significant positive correlation between ER expression (r = 0.468) and PgR (r = 0.341). Reducing the expression of ER in women who gave birth may thus reduce, among others, the expression of MCM-7 and thus explain the protective effect of past pregnancies and deliveries on the occurrence of uterine fibroids. Despite many years of research on the effects of hormonal contraceptives on uterine fibroids, there are conflicting data in the available literature. In our own study, a negative effect of the use of oral hormonal contraceptives on the expression of all tested markers was found; however, the statistical significance was confirmed only for the expression of MCM-5 and MCM-7 proteins (r = −0.326 and r = −0.447). This fact may explain the protective effect of OC on the reduction in the incidence of uterine fibroids observed by many researchers [10,16] by its influence on MCM proteins that initiate and control the course of DNA replication. In the studied cases, a positive effect of smoking on the expression of estrogen and progesterone receptors and MCM-7 protein was found (*p* < 0.05). However, this does not explain the protective effect of cigarette smoking, as observed by many researchers, that is related to the inhibition of the aromatase enzyme (an enzyme that converts androgens to estrogens), which reduces the concentration of estrogens in the studied patients and reduces the incidence of uterine fibroids [23,24]. However, in the literature, there are also studies that contradict this research; thus, the relationship between smoking and MCM protein expression requires further detailed research [13,20]. Epidemiological studies indicate that uterine fibroids may be hereditary. Patients’ questionnaires are most often used to assess the occurrence of uterine fibroids in relatives. In the studies by Templeman et al., patients were at a 1.4× higher risk of uterine fibroids than the population risk in the case of their family history (mother or sister) [63]. Similarly, Sato et al. showed as much as a fivefold higher risk of leiomyoma in patients with first-line uterine fibroids if a woman gave birth less than twice. If she gave birth more than 2×, the risk decreased to 2.1× [64]. The applied fertility criterion emphasizes the multifactorial and complex etiology of uterine fibroids and the protective effect of having offspring on the occurrence of uterine fibroids, which was confirmed in our own research. Additionally, the studies of Luoto et al. showed a more frequent presence of leiomyoma in monozygotic twins than in fraternal twins [65]. When assessing the influence of the familial occurrence of uterine fibroids on the expression of the examined MCM proteins in our own study, no statistically significant relationships were found. Only PgR expression was higher in women with a burdened history of uterine fibroids (*p* = 0.008), especially in sisters (*p* = 0.015). By using detailed immunohistochemical diagnostics, including MCM proteins, in the evaluation of uterine fibroids, a group of neoplasms with high proliferative potential could be identified. This knowledge may be useful in the preoperative evaluation of tumors of unclear clinical nature and in the prognosis of the further clinical course of these tumors in patients without clinical symptoms who do not require urgent surgery. Preoperative histopathological evaluation of uterine tumors is possible by taking a biopsy of the lesions but, due to their location, it is not always easy to perform. Kawamura et al. performed a transcervical biopsy of neoplastic lesions of the uterine muscle in 435 patients. The material obtained in this way allowed for the histopathological assessment of neoplastic changes and the proper qualification of patients for conservative or surgical treatment, including preoperative diagnosis of LMS neoplasms [66]. Assessment of the expression of MCM proteins as major regulators of DNA replication may help to determine the proliferation potential of the uterine tumors studied. Thus, preoperative knowledge of the histopathological character of the examined tumors would enable the precise planning of diagnostics and treatment, especially in clinically doubtful cases. Neoplasms originating from the smooth muscle cells of the uterus are among the most common pathological lesions of the reproductive organ. The lack of unambiguous criteria for differentiating benign and malignant uterine neoplastic changes and the lack of specific and sensitive markers of proliferation often pose a great challenge. Seemingly diagnostically trivial cases often raise doubts of the investigator, and the inability to pre-operatively assess the risk of malignancy and the possibility of further tumor growth may lead to wrong decisions regarding the method and scope of the surgery. Due to the common occurrence and the multitude of histopathological subtypes of uterine smooth muscle tumors, it is necessary to use molecular techniques in routine histopathological diagnosis of cases. Finding new markers of cell proliferation may usher in a breakthrough in the differentiation of questionable neoplastic lesions, as well as the possibility of modern therapy planning for patients.

## 5. Conclusions

The following are our conclusions:

1. Cells constituting uterine fibroids are characterized by a higher proliferative potential assessed by the expression of the Ki-67 antigen and the tested MCM family proteins compared to unchanged cells of the endometrium.
2. Due to the observed correlation of the expression of both steroid receptors, ER and PgR, with the expression of MCM-7, it seems that it is a protein of the MCM family that deserves the greatest attention.
3. Expression of the studied markers in relation to clinical data showed statistically significant relationships only in terms of obstetric history, oral contraception, and smoking habits, which suggests the possibility of using the expression of the abovementioned markers in these groups of patients.
4. The obtained results indicate the need for further research with the use of MCM family proteins in order to verify the possibility of their clinical use as a proliferative marker, which is an alternative to the routinely determined Ki67 antigen.

## Figures and Tables

**Table 1:** Clinical features of study group.

Feature	M ± SD	Me (Q_1_–Q_3_)	Min–Max
Age (year)	46.0 ± 9.2	45 (40–50)	30–75
Body weight (kg)	67.4 ± 11.0	68 (60–73)	46–100
Height (cm)	164 ± 5	164 (160–168)	153–178
BMI (kg/cm^2^)	25.0 ± 3.7	24.9 (22.6–26.9)	18.4–35.6
Date of the first menstruation (age)	13.3 ± 1.7	13.5 (13–14)	9–17
Lenght of the cycle 1 (days)	27.0 ± 3.4	28 (26–28)	16–35
Lenght of the cycle 2 (days)	30.0 ± 5.0	28 (28–30)	20–47
Lenght of the bleeding (days)	6.6 ± 2.3	6.5 (5–7)	4–14
Number of tampons (no.)	12.3 ± 8.0	10 (7–20)	5–40
The severity of menstrual pain (NRS)	4.7 ± 3.5	5 (0–8)	0–10
Pressure on the bladder (NRS)	2.6 ± 3.2	0 (0–5)	0–10
Hb level before surgery (g/dl)	12.77 ± 1.37	13.0 (11.8–13.6)	10.1–15.6
Hb level after surgery (g/dl)	11.23 ± 1.43	11.2 (10.2–12.1)	8.6–14.9
Number of pregnancies (no.)	1.8 ± 1.0	2 (1–2.5)	0–4
Number of deliveries (no.)	1.5 ± 1.0	2 (1–2)	0–3
Age at first pregnancy (age)	23.8 ± 3.4	24 (21–26)	18–33
Years since last childbirth (no.)	20.1 ± 6.8	20 (16–25)	3–31
Number of fibroids (no.)	2.5 ± 1.7	2 (1–4)	1–7
The size of the largest fibroid (cm)	5.3 ± 2.6	5 (3.5–6.5)	1–14

**Table 2:** The frequency of clinical features in the study group.

Features	*N*	%
Menstruation	35	79.5%
Regular periods	36	81.8%
The presence of blood clots	37	84.1%
Menstrual pain	31	70.5%
Analgesics	21	47.7%
Ibuprofen	16	36.4%
Paracetamol	4	9.1%
Ketoprofen	2	4.5%
Nimesulide	1	2.3%
Pain:		
Constant	12	27.3%
Before menstruation	1	2.3%
Halfway through the cycle	1	2.3%
Lack	30	68.2%
Pressure on the bladder	19	43.2%
Pollakiuria	18	40.9%
History of anemia	14	31.8%
History of blood transfusions	4	9.1%
Sterility	5	11.4%
Chronic diseases:	21	47.7%
Diabetes	2	4.5%
Hypertension	6	13.6%
Heart arrhythmia	2	4.5%
Lyme disease	2	4.5%
Hypothyroidism	6	13.6%
Gynecological diseases:	16	36.4%
Ovarian cyst	5	11.4%
Family history of uterine fibroids (medical history)	16	36.4%
Mother	13	70.5%
Sister	4	9.1%
Grandmother	3	6.8%
Nicotinism	8	18.2%
Recurrent pelvic infections	4	9.1%
Oral contraceptive pills	11	25.0%
Conservative treatment (ESMYA)	3	6.8%
Procreation plans	2	4.5%
Operations in the interview	31	70.5%

**Table 3:** Correlation between tested immunohistochemical markers.

	Ki-67	ER	PgR	MCM-3	MCM-5	MCM-7
Ki-67		r = −0.033 *p* > 0.05	r = 0.010 *p* > 0.05	r = 0.549 *p* < 0.001	r = 0.579 *p* < 0.001	r = 0.313 *p* < 0.05
ER			r = 0.693 *p* < 0.001	r = 0.114 *p* > 0.05	r = 0.195 *p* > 0.05	r = 0.468 *p* < 0.01
PgR				r = 0.130 *p* > 0.05	r = 0.172 *p* > 0.05	r = 0.341 *p* < 0.05
MCM-3					r = 0.727 *p* < 0.001	r = 0.673 *p* < 0.001
MCM-5						r = 0.687 *p* < 0.001
MCM-7						

## Data Availability

The data presented in this study are available on request from the corresponding author. The data are not publicly available due to privacy issues.
